# A Methodological Approach for Assessing Amplified Reflection Distributed Denial of Service on the Internet of Things

**DOI:** 10.3390/s16111855

**Published:** 2016-11-04

**Authors:** João José Costa Gondim, Robson de Oliveira Albuquerque, Anderson Clayton Alves Nascimento, Luis Javier García Villalba, Tai-Hoon Kim

**Affiliations:** 1 Electrical Engineering Department, University of Brasília, Campus Universitário Darci Ribeiro, 70919-970 Brasília DF, Brazil; gondim@unb.br (J.J.C.G.); robson@redes.unb.br (R.d.O.A.); andclay@ene.unb.br (A.C.A.N.); 2 Center for Data Science, Institute of Technology, University of Washington, Tacoma, WA 98402-3100, USA; 3 Group of Analysis, Security and Systems (GASS), Department of Software Engineering and Artificial Intelligence (DISIA), Faculty of Computer Science and Engineering, Office 431, Universidad Complutense de Madrid (UCM), Calle Profesor José García Santesmases, 9, Ciudad Universitaria, Madrid 28040, Spain; 4 Department of Convergence Security, Sungshin Women’s University, 249-1 Dongseon-Dong 3-ga, Seoul 136-742, Korea; taihoonn@daum.net

**Keywords:** Amplified Reflection, Distributed Denial of Service, Pentest, Risk Management, Vulnerability Assessment

## Abstract

Concerns about security on Internet of Things (IoT) cover data privacy and integrity, access control, and availability. IoT abuse in distributed denial of service attacks is a major issue, as typical IoT devices’ limited computing, communications, and power resources are prioritized in implementing functionality rather than security features. Incidents involving attacks have been reported, but without clear characterization and evaluation of threats and impacts. The main purpose of this work is to methodically assess the possible impacts of a specific class–amplified reflection distributed denial of service attacks (AR-DDoS)–against IoT. The novel approach used to empirically examine the threat represented by running the attack over a controlled environment, with IoT devices, considered the perspective of an attacker. The methodology used in tests includes that perspective, and actively prospects vulnerabilities in computer systems. This methodology defines standardized procedures for tool-independent vulnerability assessment based on strategy, and the decision flows during execution of penetration tests (pentests). After validation in different scenarios, the methodology was applied in amplified reflection distributed denial of service (AR-DDoS) attack threat assessment. Results show that, according to attack intensity, AR-DDoS saturates reflector infrastructure. Therefore, concerns about AR-DDoS are founded, but expected impact on abused IoT infrastructure and devices will be possibly as hard as on final victims.

## 1. Introduction

The exponential growth of the Internet of Things (IoT) and its widespread use in diverse areas has brought along several security issues, covering data privacy and integrity, access control, and availability, just to name the most common [1]. Typical IoT devices are, in their majority, limited in computing and communication power, so implementing security becomes an even harder task, as resources are prioritized for functionality rather than security. The general perception is that impact due to security flaws hits harder, either by device functional criticality or because of their huge numbers, or both. One major concern is the abuse of IoT infrastructure in distributed denial of service (DDoS) attacks, either to attack other infrastructures [2] or to be targeted [3,4]. There are several reports of both sorts of incidents on the Internet [5,6]. However, threats are assessed based more on inference than on factual evidence. Usually, the methods for assessing availability threats are close to stress tests. However similar in its effects, denial of service (DoS) attacks differ substantially from stress tests. The main difference is that attacks are deliberately caused by an attacker. This completelychanges the form in which DoS attack impacts are to be estimated.

In certain ways, the picture resembles what happened with universal Internet access. Then, the dissemination and use of Internet technologies brought about a dilemma: on the one hand, these technologies offer services and informational resources at low cost, such that resulting benefits cannot be ignored; on the other hand, they were not conceived with security concerns, leading to incidents involving systems compromise and information breaches.

Even before the last expansion boom in the early 1990s, security incidents in the Internet were frequent [7,8]. As the Internet grew, offering more applications (mainly e-commerce and e-government), security concerns escalated. Organizations could not refrain from using the Internet. However, they were afraid their systems could be invaded, tampered with, or attacked. Many organizations understood that an efficient form of testing and assessing risks and threats to their systems and information was to apply–under controlled conditions–the same techniques, procedures, and tools used by attackers to evaluate their systems’ level of insecurity.

There are several ways to assess the security of information systems, such as network scans used to map network topology and services, or vulnerability scans where tests could identify them. Typically, those procedures are tool-based and produce lists identifying systems and nodes, enumerating their vulnerabilities. An important difference between those procedures and Active Vulnerability Assessment (AVA) is that the latter includes penetration tests (pentests) in its repertoire. Thus, its focus is not restricted to identification and (possibly) correction recommendations, but a more thorough and precise picture. When properly conducted, it may demonstrate the impact and relative importance of vulnerabilities with respect to the organizational environment under examination.

Summarizing, AVA focuses on providing a clear vision of what finding a vulnerability during a pentest means when confronted with the organizational information security context. Those were the main motivations and concerns that led to the development of a Decision-Oriented Tool-Agnostic (DOTA) methodology for performing AVAs. DOTA emphasizes repeatability and on modeling information and decision flows along the process, regardless of tools and procedures adopted. In this last aspect, it differentiates from other methodologies, being self-contained and general, yet useful.

Back on the IoT side, the motivation is to use DOTA to test availability threats, like DDoS attacks. The objective is to assess a specific form of DDoS attack when targeting IoT devices and infrastructure. Amplified reflection DDoS (AR-DDoS) is behind most significant high bandwidth incidents from 2013 on. IoT infrastructure seems to be a good target, as it offers a large number of devices with limited capabilities and few security features. The assessment is carried out in a methodical manner using DOTA. Results obtained show that concerns about AR-DDoS are founded, but it is more likely that IoT infrastructure and devices will be hit as much as final victims.

In general terms, the main contributions presented here fall with in two categories:

1. **Methodological**: A conceptual gap present in current pentest methodologies, concerning decisional aspects in pentests is address ed and filled; DOTA–a decision-oriented tool-agnostic methodology for AVA–is presented, along with its testing and on-field real-word scenario validations and a general, tool independent, yet applicable and useful attack execution model for tests (here understood as consented and controlled cybernetic attacks) is presented.
2. **IoT Security**: The quantitative and qualitative description of amplified reflection distributed denial of service (AR-DDoS) attacks abusing IoT infrastructure and devices, and the empirical evaluation of threat surface scope and impacts.

This article is organized as follows. Section 2 presents related work, with the state of the art in terms of DDoS attacks on IoT, pentest methodologies, and the conceptual tools used in DOTA, while Section 3 describes the problem situation addressed. In Section 4, the methodological approach used in DOTA modeling is described; validation test rounds, along with their results, are reported. Section 5 discusses AR-DDoS attacks abusing IoT infrastructure and devices, where tests are executed and described along with their results. In Section 6, conclusions and further work close this paper.

## 2. Related Work

This paper involves DDoS and IoT, pentest methodologies currently available, and decision cycles, which serve as a basis for attack execution modeling. For each of them, current and relevant related work is discussed in the following.

### 2.1. DDoS Attacks and IoT

Security concerns regarding IoT are usually centered on privacy, integrity, and access control issues. Here, the focus is on availability, particularly DDoS attacks, which have been actively discussed.

Kumar, Vealey, and Srivastava [9] present a survey describing possible attacks on layers that form IoT infrastructure. Yu et al. [10] describe IoT security challenges and a possible roadmap for addressing them. Elkhodr, Shahrestani, and Cheung [11] show some of the major issues challenging the widespread adoption of the IoT, focused on the interoperability, management, security, and privacy issues in the IoT. Cvitić, Vujić, and Husnjak [12] consider IoT architectural layers and assess risks for each of them. Xylogiannopoulos, Karampelas, and Alhajj [13] use data mining techniques for early warning detection in IoT infrastructures. Yin et al. [14] developed an IoT honeypot for capturing malware.

On the specific topic of DDoS over IoT, the debate is also prolific. Arış, Oktuğ, and Yalçın [15] present a survey on DoS attacks that may target IoT environments, with the evaluation of systems that try to detect and mitigate those attacks. Pras et al. [16] argue that analysis of recent attacks shows that it is quite easy to build next generation attack tools that are able to generate DDoS attacks with a strength one thousand to one million times greater than those observed today. Sonar and Upadhyay [17] introduced a solution based on an agent to protect and deal with DDoS attacks on IoT. Zhang and Green [18] propose and test a lightweight defensive algorithm for DDoS attack over an IoT network environment. Furfaro, Malena, Molina, and Parise [19] built a simulation tool for Domain Name Service (DNS)-based AR-DDoS. Hu [20] described DDoS attacks in IoT architectures. Sgouras, Birda, and Labridis [21] evaluate the impact of DoS/DDoS attacks on smart grids’ reliability through a qualitative analysis of reliability indices based on simulation. Solis et al. [22] also evaluated the effect of a DDoS attack over a Wireless Sensor Network (WSN) over Zigbee and 6lowPAN using a network simulation environment.

#### 2.1.1. Amplified Reflection DDoS

As pointed out, DDoS attacks constitute an important issue in IoT security. Before defining and discussing AR-DDoS, some definitions are necessary, keeping in mind that the cases of interest are attacks which occur in some networked environments.

A network attack is some hostile, aggressive, or malicious action executed using the network or against its infrastructure or services. An attacker is some entity that executes an attack.

A denial of service attack (DoS) is an attack where the attacker attempts to cause partial or complete service disruption so that legitimate users cannot access the service. A DoS attack at its most basic consists of either overwhelming the service with enormous amounts of traffic or service requests, or exploits some protocol fragility, which can be motivated by design or implementation. The first are referred to as volumetric attacks, and the latter are called low volume and slow rate (low and slow) attacks.

A distributed denial of service attack (DDoS) is a DoS attack where attack action is distributed among several entities which act in coordination against the target [23]. As links increased capacity and server software matured, along with the dissemination of best practices, DoS evolved, shifting to distribution. Now, instead of a single attacker hitting the target, collections of hosts act together to that end. Higher attack efficiency required extensive preparation, since a hole architecture was necessary. The attacker had to compromise and control several hosts before the attack could be executed. Compromised hosts formed so-called zombie nets, where one host (the master) controlled the remaining hosts (the slaves), while the master was directly controlled by the attacker. This architecture provided some advantages to the attacker: attack source identification is much harder; mitigation is also much harder, as there is more then one origin. The next step in DDoS evolution was to reduce preparation effort, using malware to infect, propagate, and recruit hosts. Architecturally, compromised hosts (bots) report to a command and control server, run by the attacker [24].

An amplified reflection distributed denial of service attack, AR-DDoS is a volumetric DDoS attack where an infrastructure is abused in order to potentialize and redirect traffic to the target . Potentialization is achieved through amplification while redirection amounts for reflection. Reflection is achieved sending probes over a connectionless protocol that sends back a reply. As the protocol is connectionless, source addresses may be forged (spoofed), and the response redirected to target. Amplification occurs when the reply is larger than the stimulus probe. Apart from high efficiency and simple execution, what makes AR-DDoS attractive for attackers is that its preparation effort can be reduced to detecting potential reflectors, which are available in great number.

**Figure 1:**
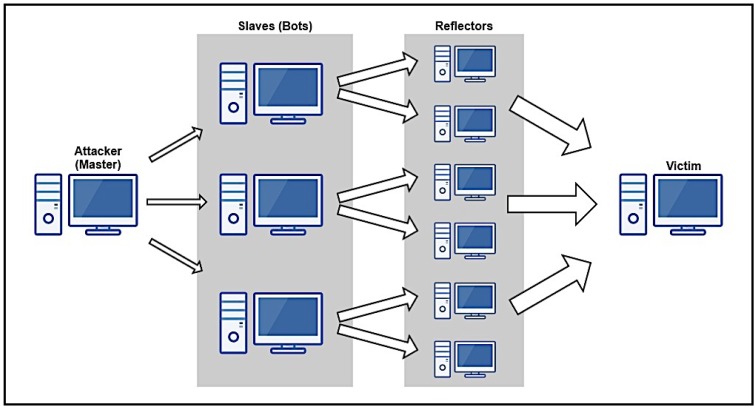
Amplified reflection attack representation.

Figure 1 illustrates a typical architecture for an AR-DDoS attack. Basically, it is identical to usual DDoS attack architecture, with reflectors before the victim to amplify traffic. As it shows, there are two layers between attacker and victim: the reflectors, which effectively send amplified traffic directly to the victim; and a set of compromised hosts (slaves or bots) which are controlled by the attacker (master) and send probes to reflectors. Rigorously, this intermediate layer is not necessary, as the attacker can efficiently perform an attack by sending probes directly to reflectors. This intermediate layer performs two functions: it contributes to increase attack scale, and provides attack source obfuscation, further protecting the attacker; besides, it is already present in the typical DDoS architecture.

In terms of attack execution, the attack master sends control information to intermediate layer hosts in order to coordinate the attack. Then, those hosts send probes crafted with the victim’s address as source address to reflectors. When a reflector receives one of those probes, it sends a properly amplified reply to the victim.

In terms of protection and defense, volumetric DDoS attacks have some well-established best practices. They can be prevented with anti-spoofing measures at access networks. Proper egress and ingress filtering rules to avoid spoofed traffic is a common best practice. However, they are not always implemented. As for mitigation, to defend against a running DDoS attack, the usual approach is to filter attack traffic as close to source as possible. The difficulty in implementing such filtering is that attack traffic normally contains spoofed source addresses. So, source address-based filtering may be inefficient, filtering the wrong source and affecting addresses that are not involved in the attack. Efficient source estimation can be achieved by analysing flows upstream of the network and then applying filters to likely sources. That, however, demands close coordination among Internet Service Providers (ISPs). There are also services that offer traffic scrubbing for a certain domain or service. Services to be protected are connected to the scrubber, which usually has great bandwidth and filtering capacity, and receives flows clean from attack traffic.

In the particular case of AR-DDoS, traffic from reflectors usually does not have spoofed source address, and source address filtering can be more precise and efficient if placed upstream.

Some protocols typically used in IoT are vulnerable to AR-DDoS. Their characteristics relevant to AR-DDoS are discussed in Section 5.1. In the case of IoT, however, there are two characteristics of the way nodes are deployed that could inherently protect them from AR-DDoS. First, a significant fraction of its networks are not connected to the internet, and incidents are reduced to insider action. The other factor is that another significant fraction of networks connected to the Internet use Network Address Translation (NAT), which hides internal topology and acts as an access filter.

Those two characteristics are bypassed in the architecture in Figure 1. By exploiting known vulnerabilities in IoT software, such as the use of unsafe login services with default credentials, an attacker can easily deploy a botnet with potentially huge numbers of bots.

In order to illustrate in practical realistic terms the kind of threat posed to IoT, two recent cases are presented. The first is a high-volume AR-DDoS attack, and the other a DDoS attack that involved over 145 thousand IoT devices.

- **300 Gbps DNS Amplification**: On March 2013 a large DNS amplification attack was directed against a renowned internet anti-spam service [25]. The incident was treated by a major traffic scrubbing facility that hosted the service [26]. In that sort of attack, amplification is achieved by abusing DNS resolvers, which are open for recursive queries. Specifically, an ANY query is sent to the DNS resolver. When answering an ANY query, the resolver replies, sending all information it has corresponding to the consulted domain. For reflection, the query spoofs the victim’s source address.
  The attack employed the usual AR-DDoS architecture. It is estimated that 30,000 unique DNS resolvers were used as reflectors. That represented around 0.1 per cent of all possible reflectors at the time of the attack. As typical DNS amplification achieves between 28 and 54 times [27], for an average factor of 40 times, the intermediate layer of bots would need to generate around 750 Mbps, which is possible with a small-sized botnet (less than one thousand bots).
  The attack started on on 18 March, with around 10 Gbps. On 19 March, it scaled to 90 Gbps, and on 22 March it reached 120 Gbps. On 27 March, the attack reached 300 Gbps of traffic related to this attack. It was the largest DDoS attack ever reported until then.
  The attack was properly handled by the traffic scrubber, with the cooperation of ISPs upstream.
- **One DDoS attack involving over 145,000 IoT devices**: On 19 September 2016, there were reports of a massive 1.1 Gbps, that settled at 900 Gbps, against an Internet hosting service [28]. Attacks lasted for over a week, with rates ranging from 100 Gbps to 800 Gbps. During the attack response, it was identified that the attack was performed by a botnet consisting of compromised hacked Internet-connected cameras and digital video recorders (DVRs), typical in smart city and smart home applications. It was estimated that each bot contributed with 1 Mbps to 30 Mbps to hit targets, with possible attack maximum capacity of 1.5 Tbps.
  Apparently, this huge collection of bots was compromised, exploiting vulnerabilities in smart devices, like default credentials and hard-coded SSH (Secure Shell) cryptographic keys.
  There were reports in the past of 25,000 Internet-connected closed-circuit TV cameras (CCTV) in DDoS attacks, and even Internet-connected refrigerators sending spam. However, traffic generated was not even close to rates reported.
  One remarkable aspect of the attack described is the fact that it did not involve amplification. That is, traffic volume was produced basically by the large number of devices compromised. It is not far-fetched to expect higher-volume attacks in the future, when amplification techniques are added.

### 2.2. Pentest Methodologies

Currently, there are several methodologies for systematizing vulnerability identification and assessment, focusing on pentesting and ethical hacking. Generally, their approach is based on operational aspects of test execution, normally involving long tool lists and detailed procedures for using them. Some of them have reduced application scope or are domain specific.

The Open Source Security Testing Methodology Manual (OSSTMM) [29] is a *de facto* standard in the pentest scenario: it is modular, almost complete, with well-defined processes and activities. However, it is complex, as it attempts to address every aspect of information security and focuses on operational aspects.

The Penetration Testing Execution Standard (PTES) [30] presents itself as a standard aimed at providing security practitioners with a common language and scope for pentest execution. It was created in 2009, motivated by a debate on pentest relevance with respect to the security market. Despite not providing technical guidelines for test execution itself, it comes with a technical guide that consists of a tool directory and techniques. Although the approach has a clear methodological bias, it is better characterized by its operational aspects and procedures for tool use, presented in the guide. Thus, despite its phase separation, the methodological aspects are shadowed by the operational appeal.

Another example of a methodology that goes along the same *rationale* is the one sponsored by the System Administration, Networking, and Security Institute (SANS) [31], which is process oriented by with strong tool orientation.

The Open Web Application Security Project (OWASP) [32] is another *de facto* standard, but its scope is restricted to web applications. Its proposal, however, is to integrate with the application development process, emphasizing post-development and pre-production. Similarly, Infosec Institute [33] presented a limited scope methodology targeting web applications.

The Payment Card Industry (PCI) Data Security Standard Council [34] also proposes a well-structured process-based methodology for commerce applications and Personal Identification Number (PIN) transaction security.

Alisherov and Sattarova [35] describe an operationally-minded methodology, specific for grid and distributed computing.

The National Institute of Standards and Technology (NIST) SP800-115 [36] is widely adopted, and it is often a compliance requirement. It is, in fact, a Risk Assessment Framework, and provides technical guidelines for information security assessment. It is operationally focused, with a defined process and detailed procedures and techniques.

The common characteristic in the methodologies reviewed is the emphasis on the procedural aspects of pentests. In some cases, procedures are presented in such a way that one is led to think that those methodologies are in fact concerned with training for pentesting, particularly in the use of tools. Those characteristics are summarized in Table 1.

### 2.3. Decision Cycles

The approach used to develop the methodology is based on a decision-making cycle generic enough to accommodate attack execution without going into specifics. In order to define this decision-making cycle for the methodology, two cycles were reviewed: *Plan–Do–Check–Act* (PDCA) and *Observe–Orient–Decide–Act* (OODA).

PDCA [37,38] is the basis for the whole framework of information security management, and is historically associated with continuous quality enhancement in quality control. The PDCA cycle clearly defines phases for planing, execution, and evaluation. However, PDCA seems more appropriate for situations where the dynamics of actions is relatively slow. Even if PDCA could serve as a foundation structuring phases in the methodology, it would not provide rapid cycles for decision and execution.

OODA, on the other hand, was conceived with fast, highly interactive, conflict environments in mind [39,40]. Decision making, right after observation, is immediately assessed. This is closer to what happens in cybernetic attacks. Even so, as it includes an orientation phase, which deals with providing options for defensive and potentially aggressive actions, where the attacker plays safe to avoid being attacked, and still preserves the ability to attack. In fact, the decision also takes simulation of what the opponent may do into account. Such level of sophistication goes beyond what is normally observed in cybernetic attacks, where attacker and defender roles almost do not alternate, especially if attacks are consented and executed under controlled circumstances.

## 3. Problem Description

Security is a deep concern in the context of computer networks and IoT, with the discussion focused on privacy, integrity, access control, availability, and resilience. With respect to availability, DDoS attacks are top of the list, either targeting IoT infrastructure and devices or abusing them to reach third parties. The consensus in the security community is that DDoS is a serious threat, and there are proposals to deal with it. However, apart from inference, qualitative and quantitative assessment of possible threat impact to IoT is needed to properly deal with them.

The usual way availability is assessed does not explicitly account for attack conditions. Therefore, if tests are to assess attack impacts, they have to include the attacker’s point of view for a more precise evaluation. This is the motivation for a methodology for vulnerability assessment which not only identifies but actively tries to exploit vulnerabilities. As reviewed in Section 2, there are several methodologies that define and guide the process of actively assessing vulnerabilities. However, despite defining processes and activities, they focus on operational aspects, and base their actions on sets of tools. The use of tools is not a problem in itself, but may reduce the scope and applicability of a methodology.

The security scenario is extremely dynamic, and a methodology coupled to a set of tools generates one of three situations: threats evolve, and new tools (along with procedures) have to be included in the methodology; defensive measures evolve, and tools either become less efficient or have to be updated, and hence the methodology too; systems evolve, and tools become obsolete, and then the need to evolve exists as in the previous cases. That is, tool obsolescence leads to methodology obsolescence. Thus, tool-based methodologies either have short life cycles or have to evolve continuously. In order to avoid this situation, a methodology should be tool-independent, or agnostic: instead of having its activities defined by the tools adopted, it should provide the means of defining the tools to be used in the process. The methodology then should focus on the *rationale* which defines actions (i.e., decisions), and should provide guidelines on what needs to be considered to decide the course of action during attack execution.

Before getting to attack execution, a series of preparatory actions is necessary: the high-level definition of what should be tested, and under which conditions; the basic information gathering about systems to be tested; attack planning from strategic to executive levels. After testing comes the reporting and presentation of results, and during the whole process, the systematic generation of records.

Those are the principles that guided the development of a decision-oriented, tool-agnostic methodology (DOTA) for AVA. DOTA formulation is intended to cover vulnerabilities in general, but is also expected to be flexible enough to accommodate not only DoS attacks, but other forms of vulnerabilities.

The IoT, along with sensor services and devices, are in the offered attack surface, and are exposed to availability threats, like denial of service (DoS) attacks. DoS attacks aim at exhausting device or infrastructure resources in order to cause service disruption, and hence its unavailability. In its volumetric form, attacks basically consist of sending a large number of requests that either overwhelm services when attempting to process them, or exceed traffic limits [41]. One common form of implementing and potentializing DoS attacks is using distribution (distributed DoS, DDoS), in which several nodes send traffic in a coordinated way, resulting in higher attack efficiency and more complex mitigation given source obfuscation.

There are several forms of DoS attacks, which can be organized in two major classes: volumetric attacks, and protocol abuse. The latter covers low volume, slow rate attacks where legitimate traffic probes exploiting specific protocol features, characteristics, or implementation details lead to an exhaustion of some of the victim’s resources, and consequently, legitimate requests are not properly responded to. The first class, on the other hand, includes attacks where large traffic volumes flood the victim, exceeding its processing capacity or link bandwidth, so that legitimate requests are not treated.

DoS attacks by protocol abuse can be broken down into protocol exploitation, when the attacker maliciously uses some feature or characteristic; and in implementation exploitation. As an example of the first modality, there is the *Slowlorris* attack, where HyperText Transfer Protocol (HTTP) GET messages are sent without completing the requested resource address, and *RUDY*, where slow rate single character HTTP POSTs are sent [42]; whereas for the second, the classical *Ping of Death* attack with oversized Internet Control Message Protocol (ICMP) datagrams is an instance [43].

As for volumetric attacks, they can also be divided into flooding attacks and amplified reflection attacks. Both forms try to overload some victim resource (usually bandwidth) by sending large traffic volumes to the victim. In flooding attacks, compromised nodes send traffic straight to the victim; while in reflection attacks, intermediate nodes–reflectors –are used to flood the victim. For the purposes of the attacker, a reflector is any node that sends an IP datagram in response to one previously received. For AR-DDoS, reflectors of interest are those that amplify; i.e., their response produces more bytes or packets, or both, than the original input datagram. This behavior is characterized by a reflection factor, indicating how much traffic is generated by the reflector. So, amplifying reflectors potentialize traffic generated by an attacker [44].

A final ingredient common to most DoS attacks, including AR-DDoS, is *IP spoofing*, which consists of crafting arbitrary source addresses in IP datagrams. In DoS attacks, attackers use this technique to obfuscate the real attack source. Characteristically, in AR-DDoS, attackers send traffic to reflectors using the victim’s address as source address [45]. The protocols most commonly abused in AR-DDoS are Domain Name Service (DNS), Network Time Protocol (NTP), Simple Network Management Protocol (SNMP), and Simple Service Discovery Protocol (SSDP). They are challenge–response, and provide significant amplification in their response [46].

### 3.1. Research Design and Description

As already presented, the consensus in the security community is that DDoS attacks pose a real threat to IoT, with its devices serving either as direct target, or for abuse in order to hit some third party.

The main purpose of this work is to assess the threat to IoT represented by AR-DDoS attacks. AR-DDoS is chosen among other forms of DDoS for its relatively low complexity and attacker effort, large availability of potential reflectors, and high efficiency. In order to fully and properly assess this threat with respect to IoT environments, it is necessary to assess impacts on reflectors and attackers, and the roles IoT devices could play in the event of an AR-DDoS attack.

The main hypothesis to be verified is related to reflector behavior, in terms of saturation. This point is crucial in assessing potential IoT abuse, since its devices would primarily be reflectors in an attack of that kind. Specifically, IoT devices’ saturation behavior when used as reflectors is to be determined. Note also that saturation may occur either as bandwidth exhaustion or excessive processing load.

Methodologically, he first option considered to characterize that sort of behavior is via network simulation. However, results would be restricted to saturation due to bandwidth exhaustion. So, simulation was not chosen.

A second option was to assess saturation behavior using stress test techniques, but that was also considered inadequate. The main reason is that those tests–however efficient in determining capacity–do not include the attacker perspective in their model; that is, the influence of a motivated entity that decides the course of actions during an attack to deliberately cause service disruption.

Implementing and running an instance of an attack (in this case, AR-DDoS) in a controlled environment was an option to be considered. With this approach, not only reflector, but also complete attack behavior could be studied. Although the idea is not completely new, this particular application is novel in the context of IoT, and could motivate interesting research questions. For example, if indeed saturation occurs, how does it influence attack efficiency; or, given overall attack behavior, how this information can be used to enhance mitigation and prevention strategies.

As a first question, there was the need to choose a particular instance of the attack. That instance should represent a realistic threat to IoT devices (see discussion in Section 5.1). There was also the requirement of fully dominating the attack cycle.

The question of choosing the attack and developing operational capabilities to perform it led to the need for a methodology–not only to guide that choice, but the whole process of planning, implementing, and executing the attack. Literature review demonstrated that there were well-established practices, but they were mostly constructed around a set of tools. As pointed out previously in Section 2.2, pentest methodologies dependent on tool sets had life cycle restrictions which might invalidate long term results.

Then, research took the path of developing a pentest methodology focusing on the decisions to guide the process. It was validated in its building blocks, and then in full rounds in different scenarios. In the sequel, this methodology was adapted to focus on availability, and later used to address the main questions of interest in this work.

The methodology guided the choice of protocol used in AR-DDoS, attack planning, implementation, and execution, which later supported result analysis and presentation.

## 4. DOTA Methodology

DOTA Methodology consists of six phases, which are executed in a sequence, each phase grouping processes and activities. For the purposes of modeling, two entities were considered: a demander and an executor. The model was produced using Business Process Modeling Notation (BPMN) [47], supported by *BizAgi Process Modeler* [48], criteriously observing the execution of each phase, which are described in the following.

### 4.1. Methodology Description

DOTA is sequential, in the sense that products of one phase are used as input for the next. DOTA phases are described and detailed bellow. For the purposes of modeling, there are two entities in the whole process: the demander and the executor. As presented in Section 2.2, current methodologies are recognized market practices, and in some cases, standards, so that DOTA follows their formatting.

#### 4.1.1. Phase 1: Task Specification

**Figure 2:**
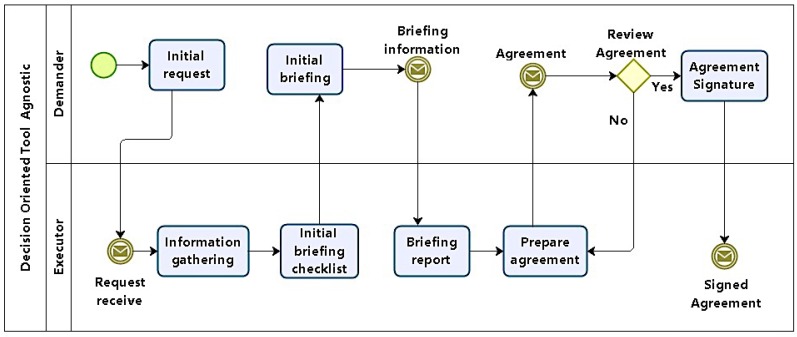
Phase 1: Task specification.

In phase 1, the task to be executed is specified. According to the process model in Figure 2, this first phase defines the conditions under which pentests will be carried out, organizing test models, scope, focus, restrictions, and constraints. It should also define how results will be treated, and to whom they will be presented. Its main products are artifacts which consolidate this agreement, including formal consent from the demanding organization.

In this phase, representatives of both demanding and executor entities will together define the focus and scope of tests. Emphasis should not be on the technical aspects involving tests, but on the strategic business importance of systems to be tested with respect to the organization. It is desirable that specific complaints concerning specific systems be reported.

Initially, a request for an AVA is placed by the demander and passed to the executor. Minimally, the demand includes a high-level definition task and main target (organization, network, host, etc.). The executor then gathers information in order to produce a checklist with questions and decisions concerning tests, which will guide the initial briefing.

The initial briefing is the moment when demander and executor answer the checklist to generate a report. The last activities in this phase involve preparing and signing an agreement between demander and executor. Although that might be unnecessary when demander and solicitor are the same, in the general case, some legal framework is needed to protect both parts.

At the end of this phase, the following questions should be answered:

1. Are tests to be executed external or internal to the demanding organization?
2. Which security property should be prioritized in tests?
3. Are there any hosts, servers, or specific addresses to be tested?
4. Is there any service–critical or not–to be tested?
5. Is there any application–critical or not–to be tested?
6. Is there any information that should be assessed in terms of confidentiality, integrity, or access control?
7. Is availability to be tested?
8. It is acceptable if tests cause denial of service?
9. Are tests to be executed furtively and stealthily?
10. What is the basic timetable, including test periods and final report presentation?
11. What are the costs and resources involved?

Answers to all questions should be mandatory. However, questions 4 to 6 may be left open, since they depend on the demanding organization’s security awareness and maturity.

#### 4.1.2. Phase 2: Initial Scans

**Figure 3:**
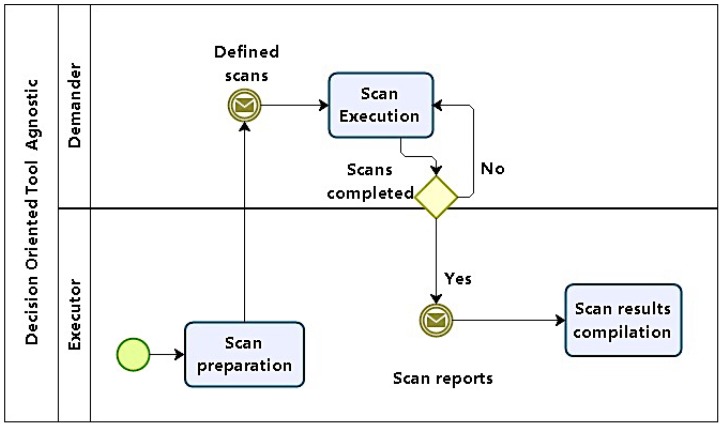
Phase 2: Initial scans.

Phase 2 starts with initial scans that will lead to the analysis of information systems and computational structures in the environment under exam in order to identify vulnerabilities (Figure 3).

Scans should be carried out in increasing levels of: depth, intrusiveness, and detail. Then, scans should start with: network sweeps to map topology and infrastructure, including not only server, but devices like firewalls and other security appliances; service scans to identify services and the software that implements them, along with their versions; and vulnerability scans targeted primarily at applications; it also should be fine tuned to cover the identified service software versions.

With information obtained in Phase 1, scans are planned and prepared. The first step is to define what will be scanned, given scope information from the initial briefing report. Execution sequence, not only in terms of networks, hosts, services, and applications (if any) to scanned, but also of the information to be obtained. Another point to be considered is whether tests are required to be performed stealthily, in order to avoid detection.

First, scans start identifying network structure and topology, then moving on to hosts. At host level, relevant information is associated with operating systems (OS) fingerprinting and service, along with server software identification. The next level focuses on finding vulnerabilities using the system and service inventory produced by previous scans. Finally, if the test scope includes applications, they are scanned.

It should be also noted that in cases such as web applications, even with available tools for scans, manual techniques involving code inspection could be necessary.

#### 4.1.3. Phase 3: Target Selection

**Figure 4:**
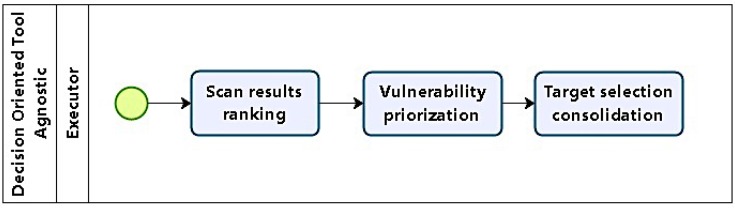
Phase 3: Target selection.

After running different scan levels, information regarding topology, services, applications, and vulnerabilities is available, and targets can be selected, using them as a basis for choice. Hosts with vulnerabilities identified in phase 2 are considered potential targets. Phase 3 (as in Figure 4) planning starts with target selection, using previous results, which are grouped and analysed. The first step is to classify and order vulnerabilities with respect to their severity, ease of exploitation, and relevance with respect to the objectives defined in phase 1.

Concerning the criteria used in classifying and prioritizing vulnerabilities, severity relates to the scope of a system’s compromising. For example, a system victim of unauthorized access which allowed restricted access to non critical resources should be ranked with low severity, but if access was granted to important or critical global resources, then the severity score should be high.

For relevance, assessment takes into account the compromised system’s functionality relative to test objectives. So, compromised hosts which do not provide services related to objectives are ranked low, whereas systems hosting relevant data are to be ranked high.

Ease of exploration relates to necessary executor skill and tool support. For example, vulnerabilities whose exploitation is supported by tools which require simple command execution are ranked high, but if there are no tools, and execution demands a series of complex procedures and involves decisions, then rank should be low.

Possible targets are consolidated in a list ordered according to the vulnerability rank, followed by relevance and ease of execution.

#### 4.1.4. Phase 4: Tactical Definitions

**Figure 5:**
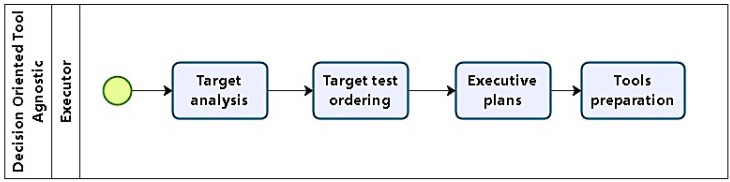
Phase 4: Tactical definitions.

Phase 4 (Figure 5) involves decisions concerning tactical definitions. With the prospective target list, exploitation methods are defined taking into account expected results and impact. Targets are also prioritized, not only in terms of importance, but also in terms of possible attack sequence, in order to have a feasible path of execution. These definitions are the basis for attack plans, which are the main artifact produced in this phase. If needed, plans may be revisited in the future in case tests do not achieve objectives.

The first step in this phase is to perform a thorough target analysis, in order to produce test plans. This contrasts with phase 3, when targets were analyzed with respect to their possible impact. Tool support is revisited to detail their availability, effectiveness, and stealthiness. Another aspect to consider is target complexity when tool support for vulnerabilities identified is inadequate or non-existent. It might be the case that some specific high-value target needs specific tools which would need to be developed, and the effort required has to be estimated.

There is also the case of emerging targets. They are low-ranked targets whose compromising in some way would facilitate the exploitation of a high-value target. Despite being non-obvious targets, their inclusion may simplify planning and reduce overall effort.

After targets are chosen and ordered, executive test (attack) plans are elaborated. Plans should include: objectives, targets (hosts and vulnerabilities involved), techniques to be used, tools (along with their configurations), and the sequence of actions with their expected results and decisions concerning the course of events.

Closing this phase, in preparation for the next, tools are prepared, including: procurement, development, installation, configuration, testing, and training (if needed), along with plan revision.

#### 4.1.5. Phase 5: Test Execution

**Figure 6:**
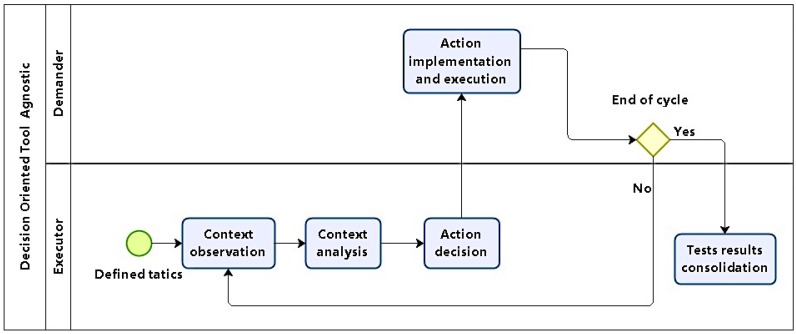
Phase 5: Test execution.

After defining each attack plan comes their execution, which will test for found vulnerabilities. DOTA models attack execution without going into the specifics of procedures, techniques, or tools, using modified OODA cycles. Thus, attack execution is modeled by *Observe–Analyze–Decide–Act* (OADA) cycles, which take advantage of certain cyber attack dynamic properties, and simplify original OODA cycles. It is in phase 5 that tests are executed, as in Figure 6.

Tests are executed as a trial and error process, where specific stimuli produce responses which may be as expected or not, having in mind that: the executor does not control targets, and scans may have produced false positive and false negative results in previous phases. By observing responses, it can be inferred whether tests were successful or not, motivating further action so that stimuli are changed to produce new responses. These on the fly adjustments tend to make revisiting phases 3 and 4 unnecessary.

Then, test plans for exploiting vulnerabilities in prioritized targets were generated. Now, plans are to be executed, and changes are included when needed as part of the expected modus operandi. As each plan is interactively carried out, test execution was modeled in cycles which adapt actions according to results obtained.

Execution cycles include four steps, as as described below:

1. **Observation**: Checking if test pre-conditions are present; when the cycle runs, the previous step post-conditions will be examined here.
2. **Analysis**: Conditions are assessed; if they are the result of some previous action, its effectiveness is evaluated, and possible corrective actions are suggested towards achieving objectives.
3. **Decision**: Given the results from previous analysis and possible actions, a choice for the next action is made.
4. **Action Implementation**: the action chosen is executed; when completed, return to observation.

Test cycles should be executed until one of the options below takes place: the objective is achieved; time runs out; or the test is aborted for some reason, such as improper planning.

#### 4.1.6. Phase 6: Reporting

**Figure 7:**
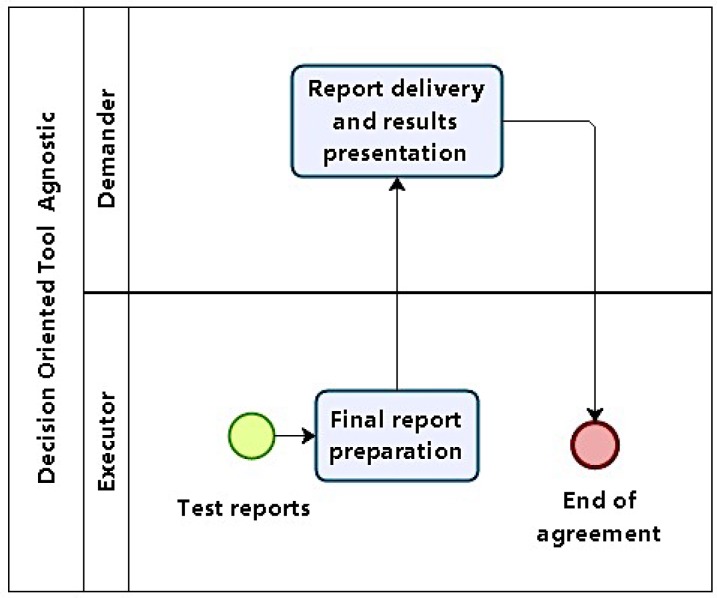
Phase 6: Reporting.

When test cycles have finished, logs and results are compiled. They will serve as a basis for reports. In phase 6, results are consolidated and presented. All previous reports, logs, and plans produced as artifacts in previous phases are grouped to produce a final report (Figure 7).

### 4.2. Results and Analysis

DOTA was developed based on practitioners, expert and industry experience. So, despite its prescriptive character, it also describes best practices. One of its goals was to produce an applicable methodology capable of generating useful results close to existing security culture.

In order to validate DOTA, the initial approach was to test isolated phases. These tests were executed in controlled environments, and led to full runs involving all phases which consolidated a first version. The next step was to test DOTA in real-world scenarios. Those tests are reported in the following.

#### 4.2.1. Test Scenario 1: Internal Infrastructure and Applications Test

The scenario for the first set of tests was a full methodology run as part of a risk assessment process executed in a medium-size organization. DOTA was run to identify and assess risks associated with the technological environment.

The summary of conditions defined in Phase 1 is presented bellow:

- The objective was to generate a panoramic view of the state of network and application security;
- There was no specific security complaint;
- The test was to be executed locally, with internal access to the network, initially without and then with user credentials;
- Scope included connectivity and network infrastructure, basic services (DNS, Simple Mail Transfer Protocol (SMTP), HTTP, etc.), and web applications;
- Tests should focus on confidentiality and integrity, as availability was out of scope;
- Two member team;
- Two weeks to test and produce report.

Under those conditions, scans were executed (Phase 2) sequentially on networks, services, and applications. During service scans, denial of service occurred repeatedly, leading to abortion of the procedure. As a consequence, scope was repactuated to exclude services.

With results from scans, targets were chosen among network devices and applications, as servers were now out of range. Network devices were prioritized, taking into account their criticality, whereas the criterion for applications was their importance and alignment with the organization’s mission. The following test plans and sequencing were defined (Phase 4). Tests were then executed (Phase 5). Results obtained were:

- All fifteen network devices, including core devices, were compromised with privilege access;
- All six web applications were compromised with several vulnerabilities (total of seventeen), including Structured Query Language (SQL) injection and Cross-Site Scripting (XSS), among others;
- Application data could be accessed and modified.

The final report was generated in about one third of the total available time. The systematic recording in previous phases–as prescribed by DOTA–had a positive impact, as reported by experts and practitioners.

The execution of a full methodology run in a production environment demonstrated that it could generate valid results in a reasonable amount of time. After presentation (Phase 6), the demanding organization was satisfied with results. The exclusion of server tests due to denial of service was also understood as a valid result (however unexpected), and indicated the need for an infrastructure upgrade. Table 2 summarizes the results in numbers.

#### 4.2.2. Test Scenario 2: External Web Application Test

The second test scenario also performed a full run of the methodology. The objective was now to assess the security of an HTTP server, along with its web applications. The methodology was executed as an independent process to identify and assess risks associated with the web site and its applications. Since the system under examination was in production, and the demanding organization did not accept service interruption during tests, the whole system was cloned in a mirror host, where tests were effectively carried out.

The execution conditions for this second test were:

- The objective was to assess server and application security;
- There were specific complaints regarding web site security;
- The test was to be executed externally to the network under exam, without user credentials;
- Scope ranged over HTTP server and web applications, both server and client side;
- Availability was out of range, and service outages were not allowed;
- Focus on confidentiality and integrity;
- Tests need not be executed furtively or stealthily, as the demanding organization’s IT team knew about them;
- Two member team;
- Four weeks to present final report.

With conditions defined (Phase 1), scans were executed–first services, then applications (Phase 2), identifying vulnerabilities. As the target was already defined, vulnerabilities to be tested were chosen from scan results (Phase 3). Vulnerabilities were prioritized according to criticality in service and application, considering their relative importance and alignment with regard to the organization’s mission (Phase 4).

After test execution (Phase 5), results were:

- Server operating system and service software was outdated with several critical vulnerabilities;
- Web application was compromised with several OWASP Top 10 vulnerabilities, some with more than one instance;
- All web application software components used (five) had critical vulnerabilities.

The final report was elaborated in about one-fifth of the available time (Phase 6), due to previous phases systematic artifact generation, as prescribed by DOTA.

The full run on a replicated production environment left testers free to test without worries about service interruption. Again, valid relevant results were obtained in reasonable time, and the demanding organization considered the results very satisfactory. Results are summarized in Table 3.

#### 4.2.3. Test Scenario 3: Comparative Test

The third scenario was the most complex, and had the purpose of providing a basis for comparing DOTA as a methodological approach as well as a methodology itself with other approaches and methodologies seen in the information security market. Another objective was to consolidate test execution modeling with OADA cycles. This test consisted of a full methodology run simultaneously with other methodologies.

The test scenario was formatted as a competition. Five teams were formed, each with five members. Teams were leveled given their relatively little exposure to pentest techniques and tools. Each team was free to adopt any methodological approach, except for one that would necessarily use DOTA.

Tests were not executed in a real production environment, but on a simulated network, each team working on an instance of it. The test was exploratory: teams received only an initial IP address, and the general task of identifying hosts, discovering vulnerabilities, and exploiting them.

Competition rules were:

- Teams were not supposed to exchange of information;
- Each team had access to an instance of the simulated environment, with the same topology, configuration, and vulnerabilities, giving them equal conditions;
- Teams did not know the environment beforehand; they only had the entry point IP address;
- Hosts in the simulated network ran distinct operating systems with distinct vulnerabilities;
- For each host identified, each team had to execute three different tasks involving vulnerability exploitation (e.g., intrusion, privilege escalation, system file modification, etc.);
- Each team had five access slots to its simulation instance, each four hours long with a 48 h interval between accesses, according to a timetable;
- After the last slot, each team had 48 h to present a report with their methods, procedures, actions, and findings;
- The team with the highest score (weighting tasks executed and report) was to be declared the winner.

The 48 h interval between accesses was motivated by limitations on available resources. However, this interval, along with the limited time slot, emphasized the need for careful action planning and optimized decisions. It must be noted that evaluating whether DOTA favored those aspects was also an objective.

**Figure 8:**
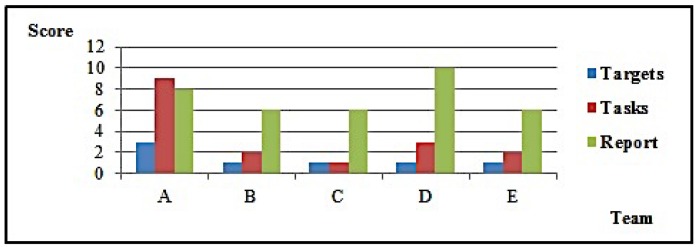
Comparative split scores–Targets, tasks, and report, organized by team.

**Figure 9:**
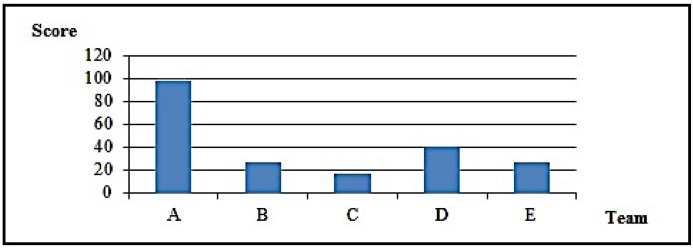
Final scores, organized by team.

All five teams followed the planned access timetable, worked according to the rules, and delivered their reports on time. Table 4 synthesizes results obtained by each team. Figure 8 and Figure 9 detail them.

Results clearly show that Team A (which used DOTA) produced results superior to those obtained by other teams, either quantitatively, by the number of targets explored and tasks executed, or qualitatively, by the form in which the whole exercise was carried out. In debriefing after tests, Team A members reported that they used the first slot only for recognizance, finding only the host with the initial address, over which they ran scans and found vulnerabilities. Then, they took advantage of the 48 h interval to plan activities for the next slot, when they managed to take over the host and find the target network. In fact, they identified not only the target network, but also the simulator management and control networks. Other teams also found those networks. However, by following DOTA, Team A kept their options open, performing wide scope scans, and found vulnerabilities in the management software that gave them the chance to completely control their simulation instance, including details of simulated host parameters. Team A then took advantage of this information and defined the sequence of host compromising and respective executive plans. Successfully, actions were performed following OADA cycles, either in test execution or in evaluating their results.

### 4.3. Synthesis

The methodology presented here was submitted to test rounds that demonstrate that its application contributes to broader, deeper, and more precise results.

During the third test, referees had to repeat tests, and the only team to have all their results confirmed was the winning one. So, the use of DOTA also contributed to the reproducibility of the results. Besides, the use of OADA cycles evidences the gains in breaking down and making explicit the decision process, not only in preparatory phases, but also in test execution.

It should be noted that between the second and third tests, there was an opportunity to evaluate DOTA flexibility. It involved security auditing a system prior to its deployment in production. The main difference in terms of applying the methodology was in Phase 2, where all system documentation was available. The effect of that difference was that, given the larger amount of relevant information about the system to be tested before test planning and execution, results were amplified; i.e., results were broader and deeper than those obtained under normal methodology application conditions.

Although it was not an original design objective (but is certainly desirable), results show that DOTA is also flexible when applied in scenarios diverse from those envisaged in its design. Table 5 shows a comparison of general scope methodologies and DOTA (OWASP was included, despite being specific to web applications, given its relevance).

Thus, even taking into account that the volume of tests is relatively small, results corroborate that DOTA application achieves objectives like repeatability, decision explicitation, and flexibility in application.

## 5. Assessing AR-DDoS Attack Threat to IoT

As described previously, security concerns in the context of IoT and sensor networks are focused on privacy and integrity. Here, focus will be on a security aspect which interfaces with areas such as network management and infrastructure: availability.

### 5.1. Using DOTA for Availability-Oriented Tests

As described in Section 4, DOTA was developed as a general methodology for active vulnerability assessment, and it was validated through its application in several different scenarios. During tests, it demonstrated its flexibility of use. Despite the fact that those scenarios focused on confidentiality and on integrity requirements, they included availability issues as test constraints. However, availability was not involved in the objectives and it was clear that DOTA application was not straightforward and needed some further considerations. Nonetheless, it was also evident that DOTA could accommodate this new scenario.

The objective now is to assess the feasibility of AR-DDoS attacks by abusing IoT infrastructure and devices. This assessment was expected to cover some quantitative and qualitative basis to realistically corroborate or refute claims about possible consequences and impact, should an AR-DDoS attack abusing IoT infrastructure take place. Particularly, a point of interest was saturation, specifically on the reflector. As a constraint, tests were to be executed in a controlled environment, so as not to attempt to cause disruption in production devices (DOTA Phase 1).

From an attacker point of view, it is necessary to choose the specific AR-DDoS attack to perform. That involves defining the protocol to be abused in reflectors. Among the protocols used in IoT devices and infrastructure, three were candidates for AR-DDoS exploitation: Constrained Application Protocol (CoAP) [49], SSDP [50], and SNMP [51] (DOTA Phase 2). All three run on top of User Datagram Protocol (UDP). The first two, CoAP and SSDP, are based on HTTP-like messages, and can provide amplification, while SNMP uses Management Information Base (MIB) variables. As for AR-DDoS attacks, there are reported incidents involving SSDP and SNMP. On the other hand, SNMP is typically used in network management, and provides the highest amplification ratio. For all three possibilities, a tool for detection and attack would have to be developed, differing only in the specific probe message to be assembled and sent. Table 6 summarizes these features.

Additionally, in terms of use, CoAP is associated with IoT, whereas SSDP and SNMP cover the scope of the Internet.

Therefore, given the high amplification rate, the large potential of reflectors in the Internet compared to other two choices and the ease of deploying a rather computationally powerful realistic IoT device network manager, the protocol chosen was SNMP (DOTA Phase 3).

The next step was to define test requirements which determine specific attack parameters to be implemented in the detection and attack tool (DOTA Phase 4). For detection, it was defined that it should not only identify the reflector, but also establish their amplification rate. For the attack, it should provide functionality to allow incremental stressing, so that saturation could be characterized. The basic plan for tests was to run the attack against a reflector that amplified and sent traffic to the target. The attack should start at a low rate and gradually escalate to higher traffic volumes. All traffic should be recorded for analysis and reporting.

On the environment side, requirements stated that in-production IoT infrastructures should not be affected. The result was the use of an isolated setup to run tests in a controlled fashion.

### 5.2. Tests, Results, and Analysis

Tests focused on amplified reflection DDoS attacks abusing SNMP. SNMP is used in network management, and is a commonly used protocol in IoT and sensor networks. AR-DDoS has been on the rise since 2013, with attacks not only more frequent, but also gradually involving higher traffic volumes [52]. What makes AR-DDoS appealing for attackers is that its requires much less preparation effort for them compared to other forms of DDoS, where nodes have to be–either manually or thorough the use of malware–compromised in preparation for attack. In AR-DDoS, attackers need only identify nodes which are vulnerable for reflection, usually due to misconfiguration. Identifying possible reflectors is easily automated in scripts and programs, which in turn are trivially modified to execute attacks (DOTA Phase 5).

#### 5.2.1. Amplification Using Simple Network Management Protocol (SNMP)

SNMP is the standard management protocol in the context of Transmission Control Protocol/Internet Protocol (TCP/IP) networks, including IoT and sensor networks. It provides mechanisms for controlled information exchange between managed and manager devices. In its first version, SNMP used *community strings* for access control. Every SNMP message had a community name which should match the accessed device community name. This simple mechanism was insufficient, since community names were transmitted in clear text, so that they were easily captured in traffic, and messages could be crafted with them. Besides, there were default community names like *public* and *private*, leading to even easier access and modification of device management information.

In SNMP version 2, operation *GetBulkRequest* was introduced to reduce request traffic between manager and managed devices, with a single request generating long replies (i.e., amplification). This operation is also present in version 3. However, version 3 security provides much stronger access control, including authentication and cryptography [53].

SNMPv2c combines operation *GetBulkRequest* with community name access control [54], as in version 1, and is still widely used. This combination of traffic amplifying operation with weak access control makes SNMPv2c easily exploitable in AR-DDoS attacks. Reference [55] shows outstanding numbers of potential SNMPv2 reflectors.

#### 5.2.2. Tool

In order to execute tests in a repeatable fashion and fully understand and control the attack cycle, a tool was developed to implement reflector discovery and the attack itself. The tool uses operation *GetNextRequest*, which queries for a single parameter for discovery, and operation *GetBulkRequest*, with values that achieve the maximum amplification rate for the device. It was developed in Java, due to its portability, and C, given its fast execution and full control over datagram crafting.

For reflector discovery over an address range, the tool sends *GetNextRequest* messages. If a reply is received, a candidate reflector is found. After exhausting the range, for each candidate reflector, the maximum amplification rate is found. The amplification factor *γ* can be expressed in either packet ($\gamma_{pkt}$) or bit amplification ($\gamma_{bit}$). $\gamma_{pkt}$ is the ratio between the number of inbound and outbound packets, $pkt_{in}$ and $pkt_{out}$, respectively, received or sent during attack (Equation 1):

(1) $$\gamma_{pkt}=\frac{pkt_{out}}{pkt_{in}}$$

while $\gamma_{bit}$ is the ratio between inbound and outbound bit flows, $bit_{in}$ and $bit_{out}$, respectively, received or sent during attack, as in Equation (2):

(2) $$\gamma_{bit}=\frac{bit_{out}}{bit_{in}}$$

*GetBulkRequest* messages are sent with a list of variables and parameters *NonRepeaters* and *MaxRepetitions*. The managed device takes the values corresponding to the first *n* variables, where n=NonRepeaters. For the remaining variables, it takes the vales corresponding to the next *m* variables, where m=MaxRepetitions. For the maximum amplification rate, a single variable value is requested NonRepeaters=0.

The choice for *MaxRepetitions* must be such that the total datagram size does not exceed 65,535 bytes. If that is exceeded (the limit for an IP datagram), there will be no response. The approach is to set the value to generate datagrams with slightly less than 65 thousand bytes. This conservative strategy is safer, since different devices store different amounts of information. So, the same value for *MaxRepetitions* sent to different devices may generate replies with different sizes. A typical maximum value for it is 2250 (see Table 7).

*MaxRepetitions* is then set by progressive approximation: an initial value is set, and the message is sent. Upon reply reception, the value is increased, and the process–transparent to the user–is repeated until reply size gets close the maximum datagram size. This approach generates more traffic, but achieves almost optimal amplification, which is a differential compared to other tools [52].

For attack, the reflectors that are to be effectively used are marked in the list of candidates, and the target defined, prior to starting execution. The tool offers eight levels of intensity. At each level, the number of probes generated is specified. It starts by generating one packet per second in level one, and at each subsequent level, the rate is multiplied by ten. With eight levels, saturation behavior is easily observed. This also satisfies the requirement that the the tool remain up to date, even with hardware or infrastructure evolution.

#### 5.2.3. Test Requirements

Tests were focused on characterizing AR-DDoS attacks quantitatively. Specific objectives were: to estimate attack effort and effectiveness; and identify saturation limits on attacker, reflector, and victim. To the best of the authors’ knowledge, there is no similar work or tool for comparing with results here.

Regarding the configuration, the reflector represents the device in an IoT or sensor network. Its configuration was deliberately chosen to be much more robust than that of typical devices in one of those networks. The reasons for this choice were: tests involved saturation, and if that occurred with small traffic, then attack behavior of interest would not be recorded; the reflector configuration is consistent with that of a network manager, including one on an IoT or sensor network, so its use is realistic; and results and conclusions hold for devices with limited computing power, bandwidth, or consumption with the proviso that saturation will occur at much lower traffic rates.

**Figure 10:**
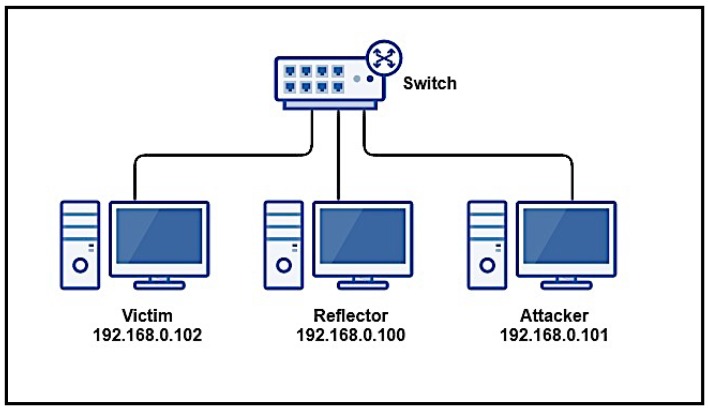
Test topology.

Tests were to be carried out in two scenarios: Test 1 and Test 2. Two different switches were used, one with low bandwidth (Switch 1 used in Test 1), and the other with high bandwidth (Switch 2 used in Test 2). The motivation for these two scenarios was to identify possible switching equipment influence in saturation results. Each scenario was a controlled environment consisting of three nodes: an attacker, a reflector, and a victim. Figure 10 illustrates test topology, where attackers correspond to a host in the intermediate layer, as in Figure 1. Nodes were connected to a switch over 100 Mbps links on a single exclusive network segment. Node configurations are shown in Table 8. For each scenario, traffic was captured on all three nodes with a protocol analyzer (Wireshark [56]). For the purposes of this test, only SNMP traffic was captured and analyzed.

Tests were executed in rounds, one for each attack intensity level. During each round, the attack was run for 30 s with traffic captured on all three nodes. Although observed, traffic related to other protocols (like ICMP) was discarded and not analyzed.

#### 5.2.4. Test Results

Test results are presented for each scenario, with Table 9a–c corresponding to Test 1 and Table 10a–c for Test 2.

Amplification factor results in both scenarios are presented in Table 11.

For Test 1, Table 9a–c show that maximum traffic (10.46 Mbps, line 3, Table 9c and 1.3 MBps, line 3, Table 9b) hits the victim at level 3, corresponding to maximum reflector output. Reflector amplification seems to start saturating from level 3 to 4 (from 864 pkt/s to 872 pkt/s, lines 3 and 4, Table 9a), while attacker saturates from level 6 to 7 (from 103 Kpkt/s to 293 Kpkt/s, Table 9a), in terms of probe generation. Attacker capacity (in terms of injected traffic) saturates between levels 4 and 5 (from 644 Kbps to 5.58 Mbps, lines 4 and 5, Table 9c and from 80 KBps to 697 KBps, lines 4 and 5, Table 9b).

For Test 2, Table 10a–c show that maximum traffic (11.02 Mbps line 3, Table 10c and 1.38 MBps, line 3, Table 10b) hits the victim at level 4, corresponding to maximum reflector output. Reflector amplification seems to start saturating from level 3 to 4 (from 875 pkt/s to 910 pkt/s, lines 3 and 4, Table 10a), while attacker saturates from level 6 to 7 (from 300 Kpkt/s to 305 Kpkt/s, Table 10a) in terms of probe generation. Attacker capacity, in terms of injected traffic, saturates between levels 4 and 5 (from 650 Kbps to 5.61 Mbps, lines 4 and 5, Table 10c and from 81 KBps to 702 KBps, lines 4 and 5, Table 10b).

Table 11 shows that for both scenarios, maximum amplification occurs at level 2, but is not sustained from that level on. Maximum amplification factors observed for Test 1 are 613.12 times in bits and 32 times in packets; while for Test 2, it was 632.18 times in bits and 33.11 times in packets. Those rates correspond to maximum attack efficiency (i.e., attack execution effort is minimal when compared to the effect on the victim).

#### 5.2.5. Test Analysis

**Figure 11:**
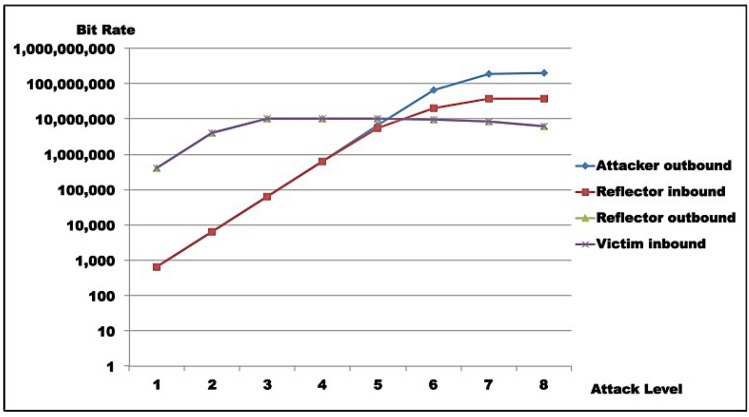
Test 1: Traffic rates by attack level (Bit/s).

**Figure 12:**
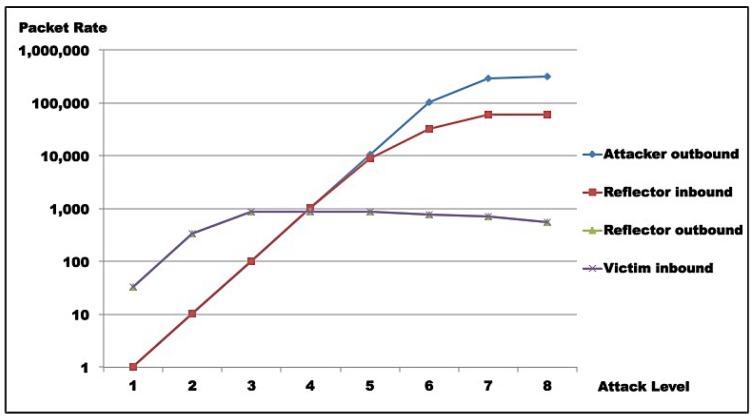
Test 1: Traffic rates (Packet/s).

**Figure 13:**
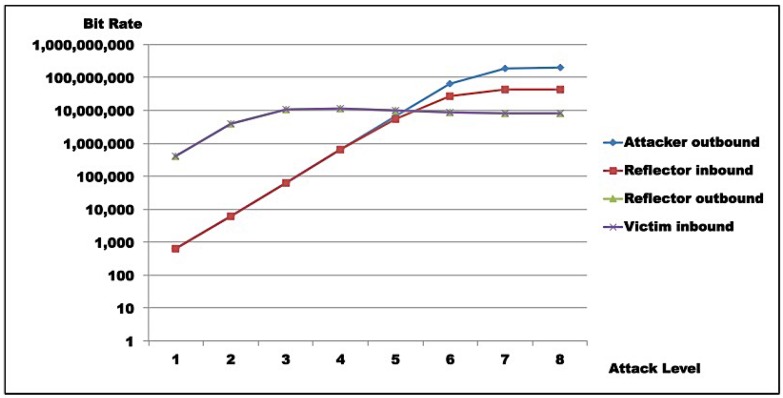
Test 2: Traffic rates by attack level (Bit/s).

**Figure 14:**
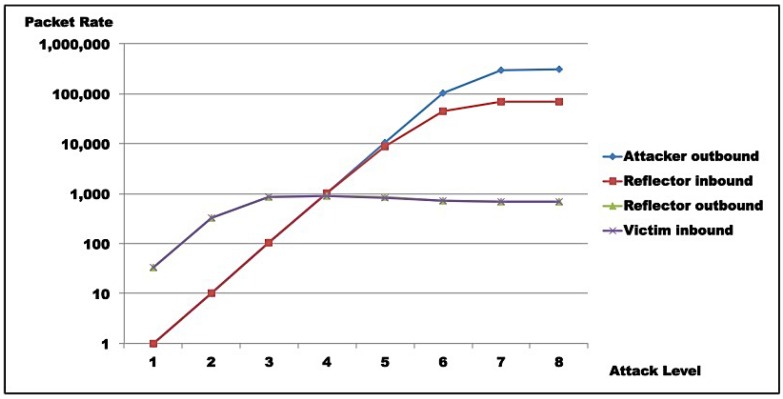
Test 2: Traffic rates (Packet/s).

Results for Test 1 are illustrated in Figure 11 and Figure 12, while Figure 13 and Figure 14 show results for Test 2. For all graphs, the horizontal axis represents attack intensity level, while the vertical axis values are according to each specific rate in logarithmic scale.

**Figure 15:**
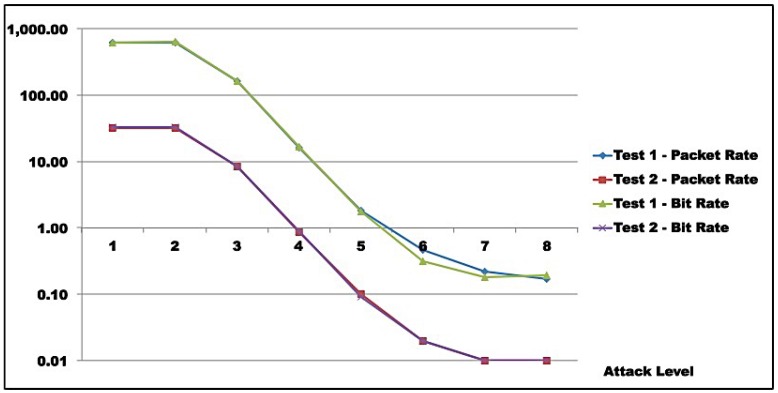
Reflector amplification factor in Bits, $\gamma_{bit}$, and Packets, $\gamma_{pkt}$, by attack level.

- **Attacker analysis**: As shown, in both scenarios, attacker-generated traffic saturates at almost 200 Mbps (198 Mbps, line 8, Table 9c and 193 Mbps, line 8, Table 10c). However, line speed is limited to 100 Mbps. This apparent inconsistency is due to the fact that the protocol analyzer used captures outbound packets prior to transmission. So, this number does not represent sent bits, but the tool’s bit generation capacity.
- **Reflector analysis**: As in the graph (Figure 11, Figure 12, Figure 13 and Figure 14), in both scenarios, reflector performance saturation appears in two forms. First, in its capacity to generate traffic, from level 3 on. It also appears in its capacity to deal with incoming traffic, around level 5. Note that graphs show that attacker outbound and reflector inbound traffic diverge at level 5, but attacker saturates generation only at level 8. So, gain is not sustainable. In fact, from level 6 on, the amplification behavior is no longer present, as the reflector sends less traffic than it receives. This occurs because *GetBulkRequest* processing is not instantaneous, as it requires information gathering and datagram assembly. As already mentioned, the reflector configuration used in these tests is much more robust than the typical networking, IoT, or sensor device. Even assuming that those devices run agents optimized for SNMP, it is still reasonable to expect that they saturate at considerably lower traffic levels.
- **Victim analysis**: The graph also demonstrates that, in both scenarios, the victim receives traffic from reflector at the rate it is sent. For successful DoS, other reflectors would be needed to either bring the victim down or flood its link.
- **Switch analysis**: In both scenarios, reflector inbound traffic is less than attacker outbound traffic. That might suggest that the switch is saturated. In fact, that motivated the use of switches with distinct capacities in each test round. However, as graphs show, reflector outbound and victim inbound traffic coincide, indicating that switches did not saturate during tests, since switch capacities largely exceed traffic volumes observed. As a whole, results in both test rounds are very close, despite different switch capacities.
- **Gain analysis**: Figure 15 shows the amplification factor in terms of packets, $\gamma_{pkt}$, and bits $\gamma_{bit}$. Both show amplification factors that are clearly not sustainable from level 2 on. From level 4, packet amplification ceases. The same happens for bit amplification from level 6 on.

Although complete DoS was not achieved in the victim, tests conclusively show that reflector saturation occurs at low injection rates. For attackers to achieve maximum amplification, careful probe generation should be exercised. For that, specific and detailed knowledge about reflectors is required.

### 5.3. Synthesis

Tests achieved their objectives and showed saturation behavior in reflector and attacker. Hence, despite obtaining high amplification rates, these are not sustainable–even for reflectors with relevant computing power. At the reflector, saturation takes place at relatively low inbound rates. The attack is feasible and effective, but needs precise execution for maximum efficiency.

## 6. Discussion, Implication, and Conclusions

### 6.1. Discussion

Test results indicate that saturation was achieved at reflector and attacker levels. In both test rounds, Figure 11, Figure 12, Figure 13 and Figure 14 indicate the reasons for saturation. For the attacker, saturation occurs when line speed is achieved. For the reflector, line speed is not achieved, and probe processing for amplification requires more processing than probe generation. So, reflector saturation is due to processing.

Results also demonstrated AR-DDoS is feasible, and can achieve high amplification rates. However, high amplification rates are not sustainable, even for reflectors with relevant computing power, as reflector saturation takes place at relatively low inbound rates. So, if attacks are not carefully conducted, the abused reflector infrastructure saturates, and victim DoS is not achieved. As a consequence, the attack would hit the reflector harder than the victim. Even so, a victim could be taken down if several reflectors coordinate their traffic. From the attacker point of view, that can be compensated by lower injection rates and more reflectors, which require more knowledge about reflector infrastructure and better attack execution management.

From the point of view of IoT and sensor networks, it has been argued that they offer expanded attack surfaces, specifically for the sort of attack described in this paper. From test results, it is clear that although the claim is realistic, the results of an attempt to abuse IoT and sensor network devices in AR-DDoS have a great chance of being confined to its own infrastructure. In any case, it is clear that IoT infrastructure and devices will be severely hit, at least as much as third party victims.

As IoT and sensor network devices and infrastructure are to be abused by reflecting and amplifying traffic, given their relatively modest computational and bandwidth capacity, reflectors will most certainly saturate, without necessarily bringing targets down. The number of reflectors involved for successful attacks will be determined by how much residual contribution each device provides in traffic directed to final victims. As observed, to take advantage of maximum efficiency, an attacker has to inject probes at low rates. By itself, that means less attacker effort, but demands higher skills and much better specific knowledge about the reflector infrastructure to be abused.

Although tests were executed over a specific implementation of AR-DDoS attacks, SNMP was chosen, as it is used in network management in IoT and has a relatively small footprint. The components that led to reflector saturation are present in most protocols candidate for abuse, namely: some computational cost for request processing, and low device computing power. If those are close, saturation will occur. So, it is not far fetched to say that conclusions presented here are fairly generalizable for AR-DDoS based on other protocols.

As a basis for comparison, Solis et al. [22] evaluates the effect of a DDoS attack over an Wireless Sensor Network (WSN) over Zigbee and 6lowPAN using a network simulation environment. Their results also indicate saturation which occurs at very low probe input rates. However, saturation is present only in terms of achieving network capacity, but there is not characterization of possible saturation due to processing load. Sgouras et al. [21] also evaluate the impact of DoS/DDoS attacks on smart grids. Saturation is also present. However, devices are not part of the attack as reflectors but victims, which is out of the scope of this work.

These results were obtained by the application of a test methodology that was flexible enough to accommodate availability threat assessment tests. The novel approach contemplates the point of view of an attacker, bringing not only another perspective, but a more complete picture regarding the chosen attack. So, a simple, easy to implement and execute DDoS attack as AR-DDoS is dissected in a series of experiments in order to characterize its traffic dynamics.

### 6.2. Implication

As presented in Section 2.1.1, incidents involving IoT devices and infrastructure are already reality, with record-breaking attacks, but amplification is not commonly used. Currently, incidents exploit simple vulnerabilities in device software, so it is more attractive to attackers to re-factor old malware to reach a huge number of new possible victims. It might be argued that efforts to enhance security in IoT should be centered in correcting faults in device software. However, that would not prevent reflection attacks. Note that if the opportunity for botnets reduce, amplification attacks over IoT are likely to become more frequent.

As for AR-DDoS prevention, current best practices are still valid. Specifically in the case of IoT, network segregation, if possible and applicable, and placement behind NAT combined with anti-spoofing filtering are simple yet efficient. However, they demand better network management and configuration control.

### 6.3. Conclusions

Presented results show that AR-DDoS is a powerful technique that enhances attack capabilities. Despite its overwhelming effects, for an attacker to make the most of it, carefully planned execution is required, contrasting with sheer brute force as in other forms of DDoS.

IoT infrastructure offers a huge attack surface in terms of AR-DDoS that has not yet been widely explored in attacks. By the saturation identified in tests, it is expected that IoT devices when used as reflectors will be hit at least as severely as victims. Fortunately, current best practices for prevention are available, and can be used to mitigate some of the attacks.

In short, the threat is real, but there are ways to deal with it; however, it requires efforts in management and enhancing IoT software.

### 6.4. Limitations

The main limitation of this work relies on the fact that the approach used is novel, not only in its application to IoT, but for availability in general. The methodology, although mature enough to generate practical useful results, still needs to be applied in different contexts. Despite that, the methodology when used correctly produces verifiable results which are applicable in real environments.

### 6.5. Future Work

This work is ongoing research which has followups in two directions: methodology, and AR-DDoS in IoT. As pointed out, the methodology used is still evolving. Experiments, not only in the scope of availability threat assessment, but active vulnerability assessment in general, to refine phases and compare with other approaches. As for AR-DDoS in IoT, a comparative study of availability threats represented by other protocols, like SSDP and CoAP, is the natural way to continue this work.

## Figures and Tables

**Table 1:** Comparative methodology chart.

Methodology	Oriented to			Scope	
	Process	Operations	Tool	Domain Specific	General
NIST	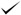	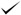	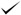		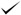
OSSTMM	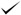	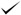	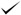		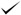
OWASP	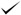	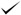	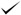	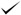	
PTES	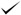	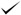	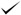		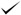
PCI	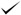	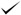	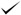	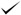	
SANS	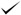	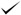	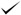		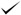

**Table 2:** Results: First test.

Scope	Targets	Vulnerabilities		
	Exploited/Total	High	Medium	Low
Infrastructure	15/15	30	15	15
Applications	6/6	9	5	3

**Table 3:** Results: Second test.

Targets	Vulnerabilities/Instances		
	High	Medium	Low
**Operating System**	1/1	3/3	2/2
**Components**	12/18	5/8	4/7
**Application**	10/13	4/4	5/8

**Table 4:** Results: Third test.

Team	Methodology	Targets/Tasks	Report	Score
A	DOTA	3/9	8	98
B	tool-based	1/2	6	26
C	tool-based	1/1	6	16
D	project oriented	1/3	10	40
E	tool-based	1/2	6	26

**Table 5:** Comparative methodology chart.

Methodology	Oriented to			
	Process	Operations	Tool	Decision
OSSTMM	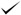	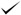	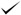	
PTES	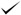	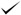	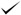	
PTI	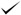	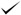	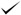	
SANS	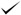	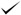	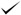	
OWASP	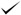	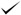	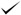	
NIST	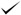	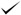	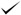	
DOTA	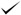			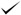

**Table 6:** Candidate protocol comparison.

Aspect	Protocol		
	CoAP	SSDP	SNMP
Uses UDP	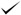	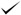	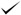
HTTP-like messages	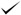	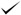	
MIB tables and variables			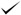
AR-DDoS reported		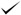	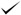
Needs tool development	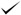	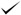	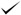
Amplification	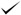	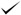	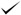

**Table 7:** *GetBulkRequest* Parameters.

Parameter	Value
*NonRepeaters*	0
*MaxRepetitions*	2250

**Table 8:** Environment configuration.

Device	Configuration	IP Address
Attacker	MAC OSX, 2.3 GHz Intel Core i7, 16 GB DDR3	192.168.0.101
Reflector	Windows 8.1 64 bits, 1.6 GHz Intel Core i5, 4 GB DDR2	192.168.0.100
Victim	Windows 7 64 bits, 3.4 GHz Intel Core i7, 8 GB DDR2	192.168.0.102
Switch 1	Multilaser E24 150 Mbps	N/A
Switch 2	Enterasys C-Series C5G124-48P2 1 Gbps	N/A

**Table:** **(a)** packet/s.

Level	Attacker	Reflector		Victim
	Outbound	Inbound	Outbound	Inbound
1	1	1	33	33
2	10	10	332	332
3	103	103	864	864
4	1021	1020	872	872
5	10,317	8835	870	873
6	103,331	32,331	785	785
7	293,869	61,129	700	699
8	314,122	60,873	547	546

**Table:** **(b)** Byte/s.

Level	Attacker	Reflector		Victim
	Outbound	Inbound	Outbound	Inbound
1	82	82	50,013	50,013
2	816	819	502,124	502,124
3	8140	8140	1,307,921	1,307,921
4	80,659	80,548	1,282,255	1,282,255
5	815,064	697,994	1,279,351	1,284,195
6	8,163,136	2,554,131	1,187,994	1,187,994
7	23,215,614	4,829,191	1,058,325	1,057,670
8	24,815,635	4,808,938	805,020	804,460

**Table:** **(c)** bit/s.

Level	Attacker	Reflector		Victim
	Outbound	Inbound	Outbound	Inbound
1	653	653	400,103	400,103
2	6531	6552	4,016,993	4,016,993
3	65,117	65,117	10,463,369	10,463,369
4	645,272	644,387	10,258,037	10,258,037
5	6,520,513	5,583,952	10,234,809	10,273,561
6	65,305,087	20,433,045	9,503,950	9,503,950
7	185,724,913	38,633,528	8,466,598	8,461,363
8	198,525,083	38,471,504	6,440,158	6,435,678

**Table:** **(a)** packet/s.

Level	Attacker	Reflector		Victim
	Outbound	Inbound	Outbound	Inbound
1	1	1	33	33
2	10	10	331	331
3	103	103	875	875
4	1031	1029	910	910
5	10,331	8889	842	839
6	103,333	44,227	717	717
7	300,173	70,229	682	682
8	305,976	70,229	685	685

**Table:** **(b)** Byte/s.

Level	Attacker	Reflector		Victim
	Outbound	Inbound	Outbound	Inbound
1	79	79	49,802	49,802
2	790	790	499,421	499,421
3	8139	8139	1,309,128	1,309,128
4	81,483	81,304	1,377,979	1,377,979
5	81,6180	702,296	1,259,074	1,254,143
6	8,163,333	3,493,969	1,075,626	1,075,626
7	23,813,690	5,548,151	1,026,244	1,026,244
8	24,172,125	5,548,164	1,033,519	1,033,519

**Table:** **(c)** bit/s.

Level	Attacker	Reflector		Victim
	Outbound	Inbound	Outbound	Inbound
1	632	632	398,419	398,419
2	6320	6320	3,995,371	399,5371
3	65,117	65,117	10,473,024	10,473,024
4	651,865	650,433	11,023,839	11,023,839
5	6,529,444	5,618,374	10,072,593	10,033,144
6	65,306,666	27,951,758	8,605,009	8,605,009
7	190,509,525	44,385,212	8,209,957	8,209,957
8	193,377,000	44,385,317	8,268,154	8,268,154

**Table 11:** Amplification rates.

Level	Test 1		Test 2	
	Bits	Packets	Bits	Packets
1	612.65	32.00	630.41	33.00
2	613.12	32.00	632.18	33.11
3	160.69	8.39	160.83	8.50
4	15.92	0.86	16.95	0.88
5	1.83	0.10	1.79	0.09
6	0.47	0.02	0.31	0.02
7	0.22	0.01	0.18	0.01
8	0.17	0.01	0.19	0.01

## References

[1] Allen N. Cybersecurity Weaknesses Threaten to Make Smart Cities More Costly and Dangerous Than Their Analog Predecessors. http://eprints.lse.ac.uk/65816/.

[2] Wueest C. (2014). The Continued Rise of DDoS Attacks. http://www.symantec.com/content/en/us/enterprise/media/securityresponse/whitepapers/the-continued-rise-of-ddos-attacks.pdf.

[3] Jackson W. (2013). How Hackers Can Turn the Internet of Things into a Weapon.

[4] Cox R. (2013). 5 Notorious DDoS Attacks in 2013: Big Problem for the Internet of Things.

[5] Sharon S. (2015). 2015 DDoS Attacks on the Rise, Attackers Shift Tactics.

[6] Toms L. (2016). Closed for Business–The Impact of Denial of Service Attacks in the IoT.

[7] Spafford E.H. (1989). The Internet Worm Program: An Analysis. SIGCOMM Comput. Commun. Rev..

[8] Stoll C. (1989). The Cuckoo’s Egg: Tracking a Spy through the Maze of Computer Espionage.

[9] Kumar S.A., Vealey T., Srivastava H. Security in Internet of Things: Challenges, Solutions and Future Directions. Proceedings of the 2016 49th Hawaii International Conference on System Sciences (HICSS).

[10] Yu T., Sekar V., Seshan S., Agarwal Y., Xu C. (2015). Handling a Trillion (Unfixable) Flaws on a Billion Devices: Rethinking Network Security for the Internet-of-Things. Proceedings of the 14th ACM Workshop on Hot Topics in Networks.

[11] Elkhodr M., Shahrestani S., Cheung H. (2016). The Internet of Things: New Interoperability, Management and Security Challenges. Int. J. Netw. Secur. Its Appl..

[12] Cvitić I., Vujić M., Husnjak S. Classification of Security Risks in the IoT Environment. Proceedings of the 26th DAAAM International Symposium on Intelligent Manufacturing and Automation.

[13] Xylogiannopoulos K., Karampelas P., Alhajj R. (2016). Real Time Early Warning DDoS Attack Detection. Proceedings of the 11th International Conference on Cyber Warfare and Security.

[14] Pa Y.M.P., Suzuki S., Yoshioka K., Matsumoto T., Kasama T., Rossow C. (2016). IoTPOT: A Novel Honeypot for Revealing Current IoT Threats. J. Inf. Process..

[15] Arış A., Oktuğ S.F., Yalçın S.B.Ö. Internet-of-Things security: Denial of service attacks. Proceedings of the 2015 23nd Signal Processing and Communications Applications Conference (SIU).

[16] Pras A., Santanna J.J., Steinberger J., Sperotto A. (2016). DDoS 3.0–How terrorists bring down the Internet. Measurement, Modelling and Evaluation of Dependable Computer and Communication Systems.

[17] Sonar K., Upadhyay H. (2016). An Approach to Secure Internet of Things Against DDoS. Proceedings of the International Conference on ICT for Sustainable Development: ICT4SD.

[18] Zhang C., Green R. (2015). Communication Security in Internet of Thing: Preventive Measure and Avoid DDoS Attack over IoT Network. Proceedings of the 18th Symposium on Communications & Networking.

[19] Furfaro A., Malena G., Molina L., Parise A. A Simulation Model for the Analysis of DDoS Amplification Attacks. Proceedings of the 17th USKSIM-AMSS International Conference on Modelling and Simulation.

[20] Hu F. (2016). Security and Privacy in Internet of Things (IoTs): Models, Algorithms, and Implementations.

[21] Sgouras K.I., Birda A.D., Labridis D.P. Cyber attack impact on critical Smart Grid infrastructures. Proceedings of the 2014 IEEE PES Innovative Smart Grid Technologies Conference (ISGT).

[22] Solis P., Pacheco L., Gondim J., Alchieri E. (2016). Evaluation of Distributed Denial of Service Threat in the Internet of Things. Proceedings of the 2016 IEEE 15th International Symposium on Network Computing and Applications (NCA).

[23] Nagpal B., Sharma P., Chauhan N., Panesar A. DDoS tools: Classification, analysis and comparison. Proceedings of the 2015 2nd International Conference on Computing for Sustainable Global Development (INDIACom).

[24] Arukonda S., Sinha S. (2015). The innocent perpetrators: Reflectors and reflection attacks. Adv. Comput. Sci..

[25] Bright P. (2013). Spamhaus DDoS Grows to Internet-Threatening Size. http://arstechnica.com/security/2013/03/spamhaus-ddos-grows-to-internetthreatening-size/.

[26] Prince M. (2013). The DDoS That Knocked Spamhaus Offline (and How We Mitigated It). https://blog.cloudflare.com/the-ddos-that-knocked-spamhaus-offline-and-ho/.

[27] US-CERT (2014). Alert (TA14-017A UDP-Based Amplification Attacks). https://www.us-cert.gov/ncas/alerts/TA14-017A.

[28] Goodin D. (2016). Record-Breaking DDoS Reportedly Delivered by >145 k Hacked Cameras. http://arstechnica.com/security/2016/09/botnet-of-145k-cameras-reportedly-deliver-internets-biggest-ddos-ever/.

[29] Herzog P. Open Source Security Testing Methodology Manual (OSSTMM). https://www.pcisecuritystandards.org/documents/PenetrationTestingGuidanceMarch2015.pdf.

[30] Penetration Testing Execution Standard: Penetration Testing Execution Standard. http://www.pentest-standard.org.

[31] SANS Institute Conducting a Penetration Test on an Organization. http://resources.infosecinstitute.com/penetration-testing-methodology-web-applications/.

[32] OWASP Testing Guide. https://www.owasp.org/index.php/OWASPTestingGuidev4TableofContents.

[33] Conducting a Penetration Test on an Organization. http://www.sans.org/reading-room/whitepapers/auditing/conducting-penetration-test-organization-67.

[34] PCI Data Security Standard (PCI DSS) Information Supplement: Penetration Testing Guidance, Version: 1.0. https://www.pcisecuritystandards.org/documents/Penetration_Testing_Guidance_March_2015.pdf.

[35] Alisherov F., Sattarova F. (2009). Methodology for Penetration Testing. Int. J. Grid Distrib. Comput..

[36] Scarfone K.A., Souppaya M.P., Cody A., Orebaugh A.D. (2008). SP 800-115. Technical Guide to Information Security Testing and Assessment.

[37] Shewhart W.A. (1939). Statistical Method from the Viewpoint of Quality Control.

[38] Deming W.E. (1986). Out of the Crisis.

[39] Boyd J.R. (1986). Patterns of Conflict.

[40] Boyd J.R. (1996). A Discourse on Winning and Losing.

[41] McDowell M. (2009). Understanding Denial-of-Service Attacks.

[42] Damon E., Dale J., Laron E., Mache J., Land N., Weiss R. (2012). Hands-on Denial of Service Lab Exercises Using SlowLoris and RUDY. Proceedings of the 2012 Information Security Curriculum Development Conference.

[43] Kenney M. (1996). Ping of Death. http://insecure.org/sploits/ping-o-death.html.

[44] Paxson V. (2001). An Analysis of Using Reflectors for Distributed Denial-of-Service Attacks. ACM SIGCOMM Computer Commun. Rev..

[45] Ali F. IP Spoofing. http://www.cisco.com/web/about/ac123/ac147/archivedissues/ipj10-4/104ip-spoofing.html.

[46] Rossow C. Amplification Hell: Revisiting Network Protocols for DDoS Abuse. Proceedings of the 21st Annual Network and Distributed System Security Symposium, NDSS 2014.

[47] Allweyer T. (2010). BPMN 2.0: Introduction to the Standard for Business Process Modeling.

[48] Transactional Process–Construction: Bizagi Process Modeler. http://www.bizagi.com.

[49] Shelby Z., Hartke K., Bormann C. (2014). The Constrained Application Protocol (CoAP).

[50] UPnP Forum UPnP Device Architecture Version 1.0, Revised on 24 April 2008. http://www.upnp.org/specs/arch/UPnP-arch-DeviceArchitecture-v1.0-20080424.pdf.

[51] Case J., Fedor M., Schoffstall M., Davin J. (1990). Simple Network Management Protocol (SNMP).

[52] Prolexic (2015). Threat Advisory: SNMP Reflection DDoS Attacks. https://www.akamai.com/us/en/multimedia/documents/state-of-the-internet/snmp-reflector-attacks-threat-advisory.pdf.

[53] Blumenthal U., Wijnen B. (2002). User-based Security Model (USM) for Version 3 of the Simple Network Management Protocol (SNMPv3).

[54] Case J., McCloghrie K., Rose M., Waldbusser S. (1996). Introduction to Community-Based SNMPv2.

[55] (2016). Open SNMP Scanning Project. https://snmpscan.shadowserver.org.

[56] (2015). Wireshark Foundation. https://www.wireshark.org.

